# Skills for adolescent WELLbeing (SWELL): protocol for a preventive effectiveness randomised controlled trial for young people at high-familial risk of depression with treatment optimisation for parents with depression at study entry comparing online group cognitive behavioural therapy (CBT) with treatment as usual

**DOI:** 10.1136/bmjopen-2025-100692

**Published:** 2025-06-19

**Authors:** Frances Rice, Victoria Powell, Olga Eyre, Rhys Bevan Jones, Daniel Michelson, Jac Airdrie, Stephan Collishaw, Anita Thapar, Detelina Grozeva, Kim Munnery, Elizabeth Randell, Lucy Brookes-Howell, Judy Garber, Ajay Thapar, Graham Moore, Neil A Harrison, Rebecca Playle, Jonathan Bisson, Rachel McNamara

**Affiliations:** 1 Wolfson Centre for Young People’s Mental Health, Cardiff University Division of Psychological Medicine and Clinical Neurosciences, Cardiff, UK; 2 Centre for Neuropsychiatric Genetics and Genomics, Cardiff University Division of Psychological Medicine and Clinical Neurosciences, Cardiff, UK; 3 National Centre for Mental Health, Cardiff University Division of Psychological Medicine and Clinical Neurosciences, Cardiff, UK; 4 Cwm Taf Morgannwg University Health Board, Abercynon, UK; 5 Department of Child and Adolescent Psychiatry, King’s College London Institute of Psychiatry Psychology & Neuroscience, London, UK; 6 NIHR Maudsley Biomedical Research Centre, London, UK; 7 Cardiff University Centre for Trials Research, Cardiff, UK; 8 Vanderbilt University, Nashville, Tennessee, USA; 9 Centre for Development, Evaluation, Complexity and Implementation in Public Health Improvement, Cardiff University School of Social Sciences, Cardiff, UK; 10 Cardiff University Division of Psychological Medicine and Clinical Neurosciences, Cardiff, UK; 11 School of Psychology, Cardiff University Brain Research Imaging Centre, Cardiff, UK

**Keywords:** Parents, Adolescent, Depression & mood disorders, PREVENTIVE MEDICINE, Psychosocial Intervention, Randomized Controlled Trial

## Abstract

**ABSTRACT:**

**Introduction:**

Young people (YP) whose parents have depression are at elevated risk for developing depression themselves and could benefit from preventive interventions. However, when parents are in a depressive episode, this reduces the effects of psychological interventions for depression in YP. Moreover, parental depression is often managed suboptimally in usual care. There is, therefore, a case for identifying and optimising parental depression treatment to enhance the effectiveness of psychological preventive interventions for depression in YP.

**Methods and analysis:**

This is a randomised controlled trial (Skills for adolescent WELLbeing) to determine the effectiveness of a cognitive behavioural therapy (CBT) intervention compared with usual care in increasing the time to a major depressive episode in YP by 9-month follow-up (primary outcome). The intervention offers a 12-week treatment-optimisation phase for parents depressed at study entry, followed by randomisation of the young person to a small group manualised online CBT programme facilitated by a therapist. YP allocated to the intervention will receive eight weekly sessions plus three monthly continuation sessions. Secondary outcomes include the number of depression-free weeks, mental health symptoms and functioning. Mechanisms of intervention action will be assessed with mediation analysis of quantitative data and thematic analysis of qualitative interviews. Participants (parents/carers with depression and their children aged 13–19 years) will be identified through existing cohorts of adults with depression, from primary care through health boards in Wales and England, UK, schools and advertising including via social media.

**Ethics and dissemination:**

The trial has received ethical approval from Wales NHS Research Ethics Committee (REC) 5, the Health Research Authority and Health and Care Research Wales (IRAS 305331; REC 22/WA/0254). This manuscript is based on V.5.7 of the protocol (17 January 2025). Findings will be disseminated in peer-reviewed journals and conferences. Reports and social media messages will be used to disseminate findings to the wider public.

**Trial registration number:**

ISRCTN13924193 (date registered: 15 March 2023).

**STRENGTHS AND LIMITATIONS OF THIS STUDY**

- This is a large-scale clinical trial of a preventive intervention, Skills for adolescent WELLbeing (SWELL), adapted from an existing evidence-based cognitive behavioural therapy manual and further developed with young people.
- The study design incorporates treatment optimisation for parents who are in a depressive episode at study entry.
- Rigorous trial methodology will be used.
- The research design does not allow the effects of parent treatment optimisation and the SWELL intervention on outcomes in the young person to be examined separately.
- For ethical and pragmatic reasons, participants are free to start any new treatment during the study period, meaning that external treatment use must be evaluated as an explanation of any differences between trial arms.

## Introduction

 Depressive symptoms and major depressive disorder (MDD) are common and often emerge during adolescence.^1^ They are associated with poor health, social and educational outcomes,^2 3^ and rates have risen sharply.^4 5^ Depression in young people (YP; aged 16–25 years)^6^ is therefore a major public health concern that requires effective, scalable prevention strategies.

In the UK, approximately 25–30% of children have a parent with treated depression.^7 8^ Having a depressed parent increases the risk of MDD by 3–10-fold in YP.^9 10^ YP with depressed parents are therefore an important high-risk group that may benefit from targeted prevention programmes.^11^

Most intervention research in this area has examined cognitive behavioural therapy (CBT) approaches.^12^,^14^ A promising example is the group-based *Coping with Stress* (CwS) programme, comprising an ‘acute phase’ (eight weekly sessions) and a ‘continuation phase’ (six monthly sessions).^15^,^17^ CwS had the largest effect size in preventing depression among YP in a Cochrane review.^12^ Moreover, these effects were relatively long-lasting (6 years).^12 17^

A US multisite effectiveness randomised controlled trial (RCT) of CwS used a combined selective and indicated prevention approach focusing on YP with parental history of depression *and* either a personal history of depression and/or elevated depression symptoms.^16^ Overall, CwS significantly reduced the risk of MDD onset in YP compared with usual care. However, a weaker main effect was found for YP whose index parent was experiencing a current depressive episode at baseline. This is consistent with other studies showing (1) current parental depression moderates CBT outcomes for depressed YP^18^ and (2) maternal depression remission is associated with decreased psychiatric symptoms and improved functioning in YP.^19 20^ Therefore, there is a case for identifying and treating parental depression to improve outcomes of preventive interventions for depressed YP.

### Study objectives

This paper describes the protocol of a study funded by the Wolfson Foundation. The study was designed to test the effectiveness of an adapted version of CwS, called Skills for adolescent WELLbeing (SWELL). The goal of the SWELL intervention is to prevent the onset/recurrence of MDD in YP at high-familial risk of depression. When designing the study, several adaptations were made with input from the trial team, the trial management group (TMG), the original intervention developers, clinicians and YP, and the potential impact of these was considered.^21 22^ The main adaptation involved including treatment optimisation for parents in a depressive episode at study entry before randomisation of the YP. This adaptation was intended to maximise the effectiveness of the intervention for YP. Although the addition of an intergenerational component might add to the burden of delivery, we judged that the potential benefits of adding parent treatment optimisation (PTO) outweighed the risk of increasing complexity of delivery.

We also made two main adaptations to the delivery of CwS. First, we reduced the number of continuation sessions from six to three. This was done with future implementation in mind, given that YP depressive symptomatology is common and access to psychological interventions is limited.^23^ As SWELL is intended to be a preventive intervention, we decided the content could be delivered effectively over a shorter period, reducing the burden of participation for YP. Indeed, a secondary analysis of CwS showed that efficacy remained with partial completion of intervention sessions.^24^ Second, we adapted the intervention for online delivery, due to scalability considerations about possible future implementation in healthcare settings. Evidence indicates that online psychological interventions are as effective as face-to-face formats,^25^ especially when this is facilitated by a therapist.^26^

In terms of presentation, we made minor updates to the materials included in the original CwS workbook. This included the use of more contemporary comic strips to illustrate key learning points. We also translated the YP workbook into Welsh to create a bilingual resource (although all intervention groups will be delivered in English during the planned trial). The adaptations described were judged necessary for a UK context with a view to future implementation in NHS settings.^21 22^

Given the amendments made to the intervention, we will carry out interviews with therapists following completion of the first two YP intervention groups and analyse with rapid content analysis.^27^ This will inform if minor procedural updates are required to ensure feasible and accessible ongoing delivery for the remainder of the trial.

### Aims

The primary aim is to test the effectiveness of SWELL in increasing the time-to-onset of YP MDD by 9-month follow-up. We will also examine the effects of the intervention on secondary outcomes including depression-free days, depression risk score, mental health problem symptoms, functioning and quality of life. A nested process evaluation will assess fidelity, adherence, the impact of adaptations on effectiveness and implementation and possible intervention mechanisms. We will undertake quantitative analyses of potential intervention mediators (eg, dysfunctional attitudes, self-efficacy) and exploratory analysis of factors that might modify effectiveness (eg, parent depression status).

## Methods and analysis

This study protocol is based on the Standard Protocol Items: Recommendations for Interventional Trials 2013 statement (online supplemental material 1).

### Trial design

This is a multisite, two-arm, parallel-group effectiveness RCT with assessors blinded to allocation. YP will be randomised 1:1 to the intervention condition (the SWELL programme plus usual care) or the usual care only condition. Given that most YP do not receive preventive interventions, usual care is the appropriate comparator. The method of randomisation will be simple-permuted-block randomisation stratified by site, with the unit of randomisation being the YP (online supplemental material 2).

### Eligibility criteria

These are outlined in box 1 and involve identifying a group of YP aged 13–19 years at high risk of depression (depression in a parent/carer plus YP current elevated symptoms/a past depressive episode). Other eligibility criteria will ensure YP can participate fully and that the parent/carer will consider engaging with PTO. We will also ensure parents do not have additional mental health diagnoses likely to impact on the appropriateness and effectiveness of PTO (eg, bipolar disorder/psychosis).^28 29^

Box 1 **Eligibility criteria**

**Inclusion criteria**

- Young person is aged 13–19 years.
- A young person is experiencing current elevated depressive symptoms as indexed by a Centre for Epidemiological Studies-Depression scale score of ≥16 and/or has a history of a Diagnostic and Statistical Manual of Mental Disorders-Fifth Edition major depressive episode but does not have a current diagnosis of depression from a doctor or health professional at screening.
- Young person is living with a parent (biological or non-biological) who has a history of depression (at least one previous episode) who will consider engaging with a depression treatment plan.
- Both the young person and parent have access to the internet via a desktop/laptop/phone/tablet and have a valid email address or mobile phone number.
- Both the young person and parent can complete trial activities as specified in the protocol, for example, sufficient understanding of the English language, absence of learning disability that would impair ability to participate and for the young person, ability to participate in small group cognitive behavioural therapy (CBT) sessions.

**Exclusion criteria**

- Young person is already receiving specialist treatment for depression (eg, currently on antidepressants or receiving CBT).
- Young person has a current diagnosis of depression from a doctor or healthcare professional.
- A young person has been told by a doctor or healthcare professional that they have a diagnosis of bipolar disorder, schizophrenia, psychosis, eating disorder or alcohol/drug dependence.
- A young person has generalised learning difficulties that, the parent judges, would prevent them from participating in trial activities.
- Parent has a diagnosis of bipolar disorder, schizophrenia or personality disorder.
- Parent is receiving treatment from secondary mental health services (eg, community mental health team or psychiatrist).
- Parent or young person not resident in the UK.

At study enrolment, if the parent is currently depressed at the screening stage, they will be offered PTO. Baseline assessments will follow completion of PTO (or immediately if the parent refuses the PTO offer), and the YP will be randomised.

### Study setting

The trial will be conducted by Cardiff University and supported by University Health Boards (UHBs) in Wales and Clinical Research Networks (CRNs) in England. UHBs and CRNs will facilitate recruitment via general practices (GPs) and mental health services. Secondary schools will also be approached to support recruitment.

### Recruitment

We will recruit via: (1) existing cohorts (https://www.ncmh.info/, https://gladstudy.org.uk/), (2) primary care and mental health services, (3) schools and (4) advertising (eg, posters/leaflets, videos) in health/community settings. Standard emails/letters/text messages with options to respond via multiple methods (study website/email/telephone) will be used to distribute study information. To help ensure a diverse sample, we intend to minimise inclusion criteria and recruit in socioeconomically diverse areas. Interested individuals will be asked to complete an online form to register interest and provide contact details. They will be contacted by telephone/videocall to complete screening with a researcher (and ask any questions). Once confirmed as eligible, the parent and YP will complete the appropriate informed consent/assent forms via phone/videocall with a researcher (online supplemental material 3). Of parents who register an interest, we estimate 25% will have a child who fulfils the eligibility criteria (ie, they have a history of depression/currently elevated symptoms) based on data from this population.^30^,^32^ Recruitment progress will be monitored regularly against expected targets.

### Changes to the trial since recruitment began

In consultation with the study TMG and trial steering committee (TSC), minor changes were made to broaden inclusion criteria to better reflect how the intervention would be delivered in practice (online supplemental material 4; Analyses).

### Sample size

We aim to recruit 400 YP and parents/carers, based on a previous study^17^ and sample size calculations. We allow for 10% loss to follow-up based on previous studies of this population and depressed YP.^1630^,^32^ The effect size observed in the study by Garber *et al*^16^ was a HR of 0.63. We expect the effect of the intervention in this RCT to be slightly greater than this due to the addition of PTO before randomisation. We therefore define the minimum clinically important difference for this study as an HR of at least 0.60 (equivalent to an 11% difference in MDD between study arms). With a sample size of up to 400 randomised participants, we estimate statistical power as 79.9% to detect a difference of at least HR=0.60 between study arms, with an assumed 10% attrition rate at 9 months, expected event rate probabilities at follow-up of 0.327 (control) and 0.211 (intervention) based on^17^ a one-sided alpha of 0.05. A HR of 0.56 (equivalent to a 12% difference between study arms) will attain 87.0% power. A STATA V.17 programme was used for this estimation.^33^

### Blinding

Blinding therapists or participants is not possible for the intervention under study. Assessors at baseline and follow-ups will be blind to the intervention arm. Participants will be reminded of the importance of assessors remaining blind to allocation as recommended,^34^ and assessors will report any instances of potential unblinding. The trial statistician will remain blinded to group allocation during analysis. It will not be possible to blind the qualitative analyst(s). It is not possible to blind YP, which could lead to reporting bias. We will mitigate this by using an interviewer-led criterion-based clinical interview to define the primary outcome and standardised prompts for questionnaires. We will also evaluate the consistency of effects for outcomes reported by different raters (YP and parents/carers).

### Interventions

#### Parent treatment optimisation

The rationale of PTO is to provide good quality healthcare with the aim of improving the effect size observed previously for the YP intervention.^16^ Parents meeting the threshold for depression at screening (defined as a Patient Health Questionnaire-9^35^ score≥10) will be offered up to 12 weeks of PTO. We expect 40% of parents to meet this criterion.^16 30^ Box 2 lists PTO components based on evidence-based practice.^31^ In brief, an initial assessment by a psychiatrist with recommendations to optimise depression treatment including psychoeducation, referral to online guided CBT and medication review (box 2). Fortnightly check-ins by a psychiatrist/psychology assistant will monitor progress. The duration of PTO was based on adult depression treatment trials where most individuals who remit/respond do so within 10–12 weeks^36^,^38^ and to allow the intervention for YP (delivered after PTO) to be delivered within projected timelines. The treatment team includes consultant trial psychiatrists (RBJ, NAH) at Cardiff University Psychiatry Service (CUPS) and psychology assistants. A team member will contact parents who screen positive for depression to arrange appointments (in person at the CUPS clinic or video/phone call; usually via videocall) and the parent’s GP will be informed.

Box 2 **Components involved in parent treatment optimisation**

**Initial consultation**

1. Assessment: parent is assessed by the psychiatrist in a 1-hour consultation (approximately) conducted in person or via video/phone call.
2. Psychoeducation: brief psychoeducation about depression and self-management advice (eg, activity scheduling, lifestyle approaches) will be delivered by the psychiatrist at the end of the initial consultation.
3. Guided online cognitive behavioural therapy (CBT): psychiatrist signposts the parent to the 8-module ‘Space from Depression’ or ‘Space from Depression and Anxiety’ programmes on SilverCloud platform (https://pthb.nhs.wales/services/adult-and-older-peoples-mental-health-services/silvercloud-online-cbt/)^36 48^ or equivalent. They will be advised by the psychiatrist to complete one module (roughly 1 hour each in length) per week, as recommended.^49^ The SilverCloud online CBT package will be guided as this increases adherence and response for those with moderate depression.^28^
4. Medication review: psychiatrist to review the parent’s medication history and make recommendations to the general practices (GP) regarding the medication and other aspects of the management as required.

**Follow-up consultations**

1. According to the needs of the individual as assessed by the psychiatrist, either the psychiatrist or a psychology assistant checks in with the parent once every 2 weeks in person or via video or phone call to check on their progress for up to 12 weeks. Ahead of each check-in, a reminder will be sent (eg, via text or email) to the parents with a link to complete a Patient Health Questionnaire-9 (PHQ-9)^35^ questionnaire to assess their depressive symptoms.

All parent treatment optimisation will involve assessment and signposting to guided internet-based CBT with regular follow-up. However, not all components or steps need to be completed for every parent and will be provided depending on the needs of the individual. The psychiatrists are highly trained and will use their clinical judgement regarding the treatment approaches. Therefore, there are no pre-specified escalation protocols, but typically the treatment would be escalated if there is no improvement in symptoms or if there is a deterioration in symptoms or clinical concern (eg, risk of self-harm or suicide).

#### SWELL programme

YP in the SWELL intervention arm will be assigned to an online group including 6–8 YP and a therapist. The group will meet via videocall for 60 min. Table 1 outlines the sessions which target factors hypothesised to contribute to the development/maintenance of depression. These include training in cognitive-restructuring skills and techniques for modifying cognitive, problem-solving, behavioural activation, assertiveness and relaxation. Continuation sessions involve revising and consolidating learning, planning how skills could be used in response to future stressors and troubleshooting attempts to apply skills. YP are provided with a workbook to help them follow the intervention content and apply the skills. Two parent information sessions are offered.

**Table 1:** Overview of Skills for adolescent WELLbeing (SWELL)

Acute session 1	'Beginnings’Psychoeducation about stress and depression, identifying personal goals and measuring your mood.	Intervention components common to each sessionSession start: Ice breaker. Home practice review (excluding session 1). Mood check-in with prompts to consider contributory internal and external factors. Session end: Participants encouraged to share positive events that occurred during the previous week. Participants were encouraged to reflect on aspects of the session they found most helpful and to provide feedback on what could be different. Home practice is set related to applying learning from the content of the session in their day-to-day lives.
Acute session 2	'Coping with stress’Identifying negative automatic thoughts and triggering events.	
Acute session 3	‘Increasing positive and balanced thinking’Increasing positive and balanced thinking, review of personal goals.	
Acute session 4	‘Examining negative thoughts and sources of beliefs’Identifying unrealistic thoughts and underlying beliefs, changing these to more realistic thoughts.	
Acute session 5	'Problem solving’Problem solving – thinking of solutions, evaluating solutions, trying out solutions and evaluating their effects.	
Acute session 6	‘Being Assertive’Assertiveness training, communication styles.	
Acute session 7	'Behavioural activation’Behavioural activation and integrating into daily life (plans for action and developing positive habits).	
Acute session 8	Relaxation and ending the weekly MeetingsRelaxation techniques, progressive relaxation, pleasant imagery, breathing exercises.	
Parent information session 1	A 60-minute session during week 1 of the acute session phase. Includes details about the practicalities of the young person attending the group, theory underlying the intervention, how parents can support young people to engage with home practice activities between sessions and an overview of the content of each session. Focuses on the content of sessions 1–4.	
Parent information session 2	A 60-minute session during week 4 of the acute session phase. As above but focuses on the content of sessions 5-8 and the continuation sessions.	
Continuation session 1	Emergency planning, maintaining gains.	
Continuation session 2	Review of cognitive restructuring, introduce thought-stopping techniques.	
Continuation session 3	Review of behavioural/emotion-focused content (problem solving, behavioural activation, assertiveness training).	
Therapist training and ongoing quality assurance	Training in SWELL includes 5 days of structured teaching on the underlying theory and rationale and practical delivery of the intervention including training in non-specific psychotherapeutic skills (eg, active listening, Socratic questioning) and running therapeutic groups (eg, managing group dynamics). In addition, therapists will observe the delivery of the intervention by the clinical psychologist before being observed delivering it themselves. Therapists’ competency delivering SWELL will be assessed using an adapted version of the Assessment of Core CBT Skills (ACCS^50^). Therapists will require a rating of “Good” in all domains before being approved to deliver the intervention independently. On completion and sign-off of therapists to deliver the intervention independently, an early (session 1–4) and late (session 5–8) will be selected at random (using a random number generator) to be listened to and rated by the therapist, and independently rated by a clinical psychologist using the ACCS, with feedback provided during monthly individual supervision sessions with particular focus on areas rated as less than ‘Good’. In subsequent supervision sessions, close attention will be paid to areas rated less than ‘Good’ in a previous session to ensure therapists are able to rectify any issues. If ongoing issues are identified, further training will be provided.	

CBT, cognitive behavioural therapy.

Each group will be led by a trained therapist, with a psychology/related degree, experience of delivering psychological interventions to YP and knowledge of CBT. A secondary group facilitator will monitor the group chat and provide technical help. Therapists will follow the SWELL intervention manual. As outlined earlier, we consulted with the original CwS intervention developers in the adaptation process to ensure core elements of the original intervention were retained. Table 1 outlines therapist training and quality assurance measures. Training and fortnightly supervision (alternating between group and individual supervision) will be delivered by an experienced clinical psychologist (JA).

#### Usual care

Participants in both trial arms will be able to continue with any existing treatment consistent with the eligibility criteria and will also be permitted to begin any new treatments during the study. Treatment received by YP and parents will be recorded at 9 months using the Client Service Receipt Inventory.^39^

### Adherence

Adherence to the intervention for YP will be defined as attending 6 out of 8 acute sessions and 2 out of 3 continuation sessions. Adherence will be rewarded by providing certificates following completion of the programme. Therapists will also offer sessions scheduled outside the school day and one-to-one catch-up sessions if YP are unable to attend a session. Attendance for each participant will be monitored. For PTO, the opportunity to be assessed and treated by a specialist (psychiatrist) may be attractive, and we will offer flexibility in how parents complete appointments (in person/videocall/phonecall). Adherence to PTO will be defined as completion of the baseline assessment and four follow-up calls.

### Retention

Participants (parents and YP) will each receive £60 in gift vouchers for participation (£10 at baseline, £20 at 3 months, £30 at 9 months) and study completion certificates at 3 months and 9 months. Those who also participate in qualitative interviews and/or focus groups will receive an additional £20 voucher.

### Patient and public involvement

Adaptations to the intervention were made following discussions with the TMG, the CwS intervention developers, professionals and our study advisory group of 12 YP with lived experience of depression/anxiety (YPWLE). The latter advisory group contributed feedback on protocol development during three meetings (two online, one hybrid) and a smaller group contributed on one additional occasion. YPWLE advised on: the study name, assessments, timings of intervention/assessments and adaptation of the intervention (workbook content including language, visuals, online delivery methods). One young person and a parent with experience of depression are members of the independent TSC.

### Outcomes and assessments

**Figure 1:**
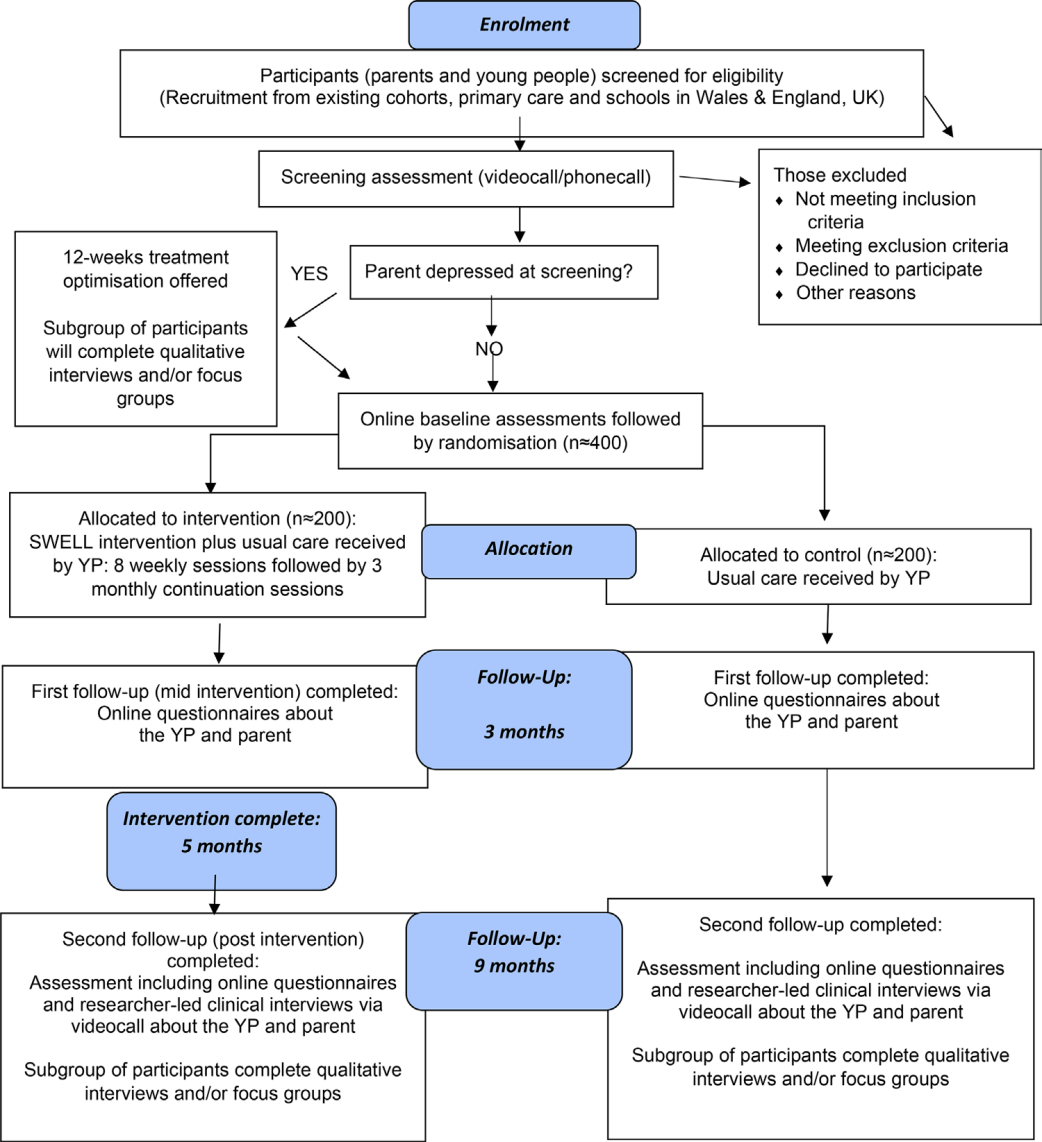
Participant timeline. SWELL, Skills for Aadolescent WELLbeing; YP, young people.

Parents and YP will complete assessments with a researcher via videocall at initial screening, at baseline and 9-month follow-up post randomisation. Self-report questionnaires will be completed at baseline, 3 months and 9 months (figure 1). We selected 9-month post randomisation to assess the primary outcome for consistency with prior studies^16^ and because this aligns with completion of the full intervention. The 3-month postrandomisation follow-up was selected to allow for measurement of potential mediators and secondary outcomes following completion of the acute intervention sessions. Table 2 shows the schedule of measures.

**Table 2:** Schedule of assessments

	Follow-up			
	Screen	Baseline	Month 3	Month 9
Questionnaires about young people				
Eligibility questions (eg, psychiatric history and treatment)	P			
Centre for Epidemiological Studies Depression Scale	Y	Y	Y	Y
Demographic characteristics (eg, age, sex, ethnicity, education)		Y, P		
Revised Children’s Anxiety and Depression Scale		Y, P	Y, P	Y, P
Affective Reactivity Index		Y, P	Y, P	Y, P
Social Communication Disorders Checklist		P		
DuPaul ADHD Questionnaire		P		
Dysfunctional Attitudes Scale for Children		Y	Y	Y
Generalised Self-Efficacy Scale		Y	Y	Y
Perceived Stress Scale		Y	Y	Y
Behavioural Activation Scale for Depression- short form		Y	Y	Y
Developmental Competence Scale (abbreviated)		Y		Y
Conflict Behaviour Questionnaire		P	P	P
Child Report of Parents Behaviour Scale		Y	Y	Y
Parental monitoring (child disclosure questions)		P	P	P
Life Events Checklist		Y, P		Y, P
EQ-5D-Y		Y		Y
Young person’s experience of the intervention			Y, P	
Interviews about young people				
Development and Well Being Assessment		Y, P		
Client Service Receipt Inventory		Y, P		Y, P
Longitudinal Interval Follow-Up Evaluation				Y, P
Questionnaires about Parents				
Eligibility questions (eg, psychiatric history and treatment)	P			
Patient Health Questionnaire-9	P	P	P	P
Demographic characteristics (including age, sex, ethnicity, education level, income, occupation)		P		
Screen for Adult Anxiety Related Disorders		P	P	P
WHO Disability Assessment Schedule		P		P
Parent’s experience of the parent treatment optimisation		P		
Interviews about parents				
Schedules for clinical assessment in neuropsychiatry		P		
Psychiatric history, using life history calendar approach		P		
Client Service Receipt Inventory		P		P
Longitudinal Interval Follow-Up Evaluation				P
Other Measures				
Depression risk score		Int		Int
Children’s Global Assessment Scale		Int		Int
Global Assessment of Functioning		Int		Int
Hamilton Depression Rating Scale		Int		Int
Parent treatment optimisation adherence checklist		Res		
Young person intervention adherence checklist				Res
Young person intervention fidelity checklist			Res	

In addition to completing these measures, young people and their parents will be asked for consent to data linkage with routine healthcare data (in Wales this is via the Secure Anonymous Information Linkage databank). This will enable further follow-up of study participants to examine longer-term outcomes including depression diagnosis and treatment.

ADHD, Attention Deficit Hyperactivity Disorder; EQ-5D-Y, EuroQol Quality of Life 5 Dimensions; Int, completed by interviewer; P, parent completed; Res, member of the research team; Y, young person completed.

#### Primary outcome

##### Depressive episodes

The primary outcome is the time to a probable Diagnostic and Statistical Manual of Mental Disorders-Fifth Edition^40^ MDD episode in the young person during the 9-month follow-up period. We will collect data about when any depressive episode occurs during the follow-up period using the LIFE,^41^,^43^ a semistructured interview, that uses a life-calendar approach allowing diagnoses and the week-of-onset to be derived. For each week of follow-up, symptoms are rated using the six-point Depression Symptom Rating (DSR) scale, which has good reliability and validity.^43^ A DSR score of >4 for at least 2 weeks will define a depressive episode.^16^ YP and parents will be interviewed with the LIFE about the YP’s depression, with parent and YP reports combined at the diagnosis level consistent with clinical practice.

### Secondary outcomes

These assess the potential effect of the intervention on depression severity and recovery, dimensional measures of mental health problems (depressive symptoms, anxiety symptoms, irritability), depression risk score,^44^ quality of life, functioning and competence. These will be measured using standardised and validated questionnaires/interviews (box 3).

Box 3 **Description of secondary outcome measures**

Depression-free periods during the 9-month follow-up

- Number of depression-free weeks, the duration of depression-free periods, time to recovery from depressive episodes and time to recurrence of depressive episodes in the 9-month follow-up period will be measured using the Longitudinal Interval Follow-Up Evaluation (LIFE). 41,43 This semistructured interview (also used to assess the primary outcome) assesses the longitudinal course of depression in enough detail to date individual episodes, allowing calculation of time to recovery, length of depression-free intervals and time to relapse or recurrence. Young people and parents will complete the LIFE about the young person’s depression.

Depression symptoms

- Will be measured using the Centre for Epidemiological Studies-depression scale (CES-D)^51^ completed by the young person. This measure includes 20 items about symptoms in the past week and has good internal consistency across clinical (α=0.83–0.84) and non-clinical samples (α=0.81–0.93).^52^

Depression Risk Score

- A Depression Risk Score tool^44^ will be used to assess if the intervention affects the young person’s 3-year risk of developing depression. This score has been developed and externally validated in young people with a parent with a history of recurrent depression.^44^ The risk score is based on age, sex, family income, young person’s symptoms of anxiety, depression and presence of stressful life events in the past 9 months, plus current parent depression symptoms.

Anxiety symptoms

- The Revised Children’s Anxiety and Depression Scale (RCADS)^52 53^ will be used to measure anxiety symptoms and will be completed by both the young person and their parent.

Irritability symptoms

- Irritability will be measured using the Affective Reactivity Index (ARI).^54^ The ARI is a dimensional measure of irritability containing seven items scored on a 3-point scale and will be completed by young people and their parents.

Quality of life

- Will be assessed using the young person report of the EuroQol Quality of Life 5 Dimensions (EQ-5D-Y) which consists of questions about mobility, looking after self, doing usual activities, pain/discomfort, and feeling worried, sad, or unhappy,^55^ plus a vertical visual analogue scale ranging from ‘the best health you can imagine’ to ‘the worst health you can imagine’.

Developmental competence

- A modified version of the scale used by Brent *et al*^17^ will be completed by the young person. The scale assesses developmental competence (including academic and interpersonal competence) and includes 9 items scored 0–4.

Functional impairment

- The Children’s Global Assessment Scale (cGAS)^56^ will be completed by the research interviewer about the young person’s functioning. The cGAS measures psychological and social functioning rated on a scale of 0–100, with a lower score indicating greater impairment.

CES-D was selected as the measure of depressive symptoms for consistency with.^16^ The RCADS was additionally included to assess anxiety, and because it is recommended as a common measure of anxiety and depression (https://iamhrf.org/projects/driving-adoption-common-measures).

### Mediators

**Figure 2:**
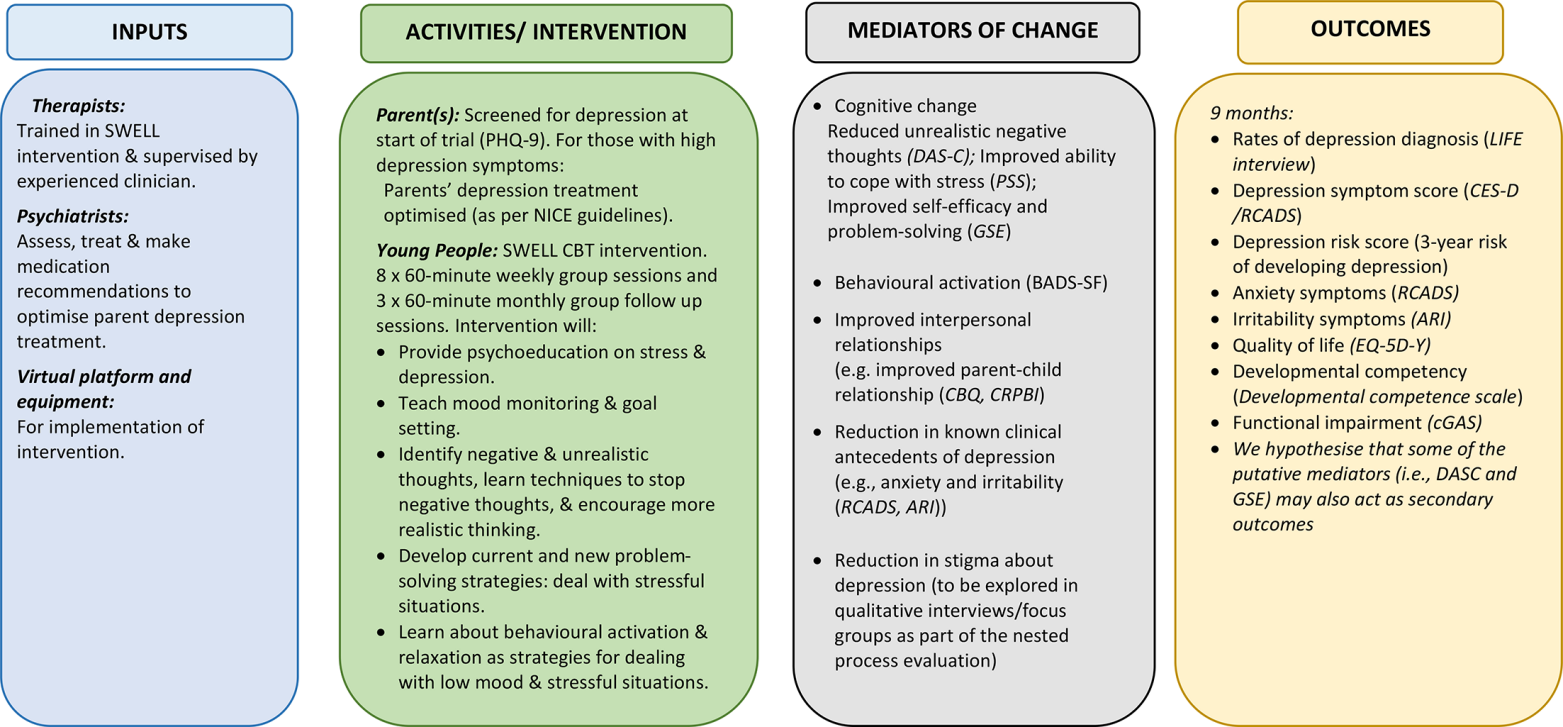
Logic model of SWELL intervention on depression, mental health and functioning in the young person. ARI, Affective Reactivity Index; BADS-SF, Behavioural Activation Scale for Depression - short form; CBQ, Conflict Behaviour Questionnaire; CBT, Cognitive Behavioural T﻿Cognitive Behavioural Therapy; CES-D, Centre for Epidemiological Studies-depression scale; cGAS, Children’s Global Assessment Scale; CRPBI, Child’s Report of Parent Behaviour Inventory; DAS-C, Dysfunctional Attitudes Scale for Children; EQ-5D-Y, EuroQol Quality of Life 5 Dimensions; GSE, Generalised Self-Efficacy Scale; LIFE, Longitudinal Interval Follow-Up Evaluation; NICE, National Institute for Health and Care Excellence; PHQ-9, Patient Health Questionnaire-9; PSS, Perceived Stress Scale; RCADS, Revised Children’s Anxiety and Depression Scale; SWELL, Skills for adolescent WELLbeing.

The logic model (figure 2) shows hypothesised mediators of change for the intervention. These include measures of cognitive change, behavioural activation, perceived coping efficacy, perceived stress and quality of the parent-child relationship indexed by warmth, conflict and openness. Mediators are either targeted by the intervention; evidence indicates they may contribute to the onset and maintenance of depression and/or index environmentally mediated intergenerational links between parent and YP depression.^1645^,^47^ Possible mediators will be measured at baseline, 3 months and 9 months post randomisation. Qualitative interviews will be conducted to explore participant perceptions of possible mechanisms of action and non-specific mediators such as perceptions of the possibility of change and the group/therapeutic alliance.^45^

### Additional measures

Table 2 includes baseline measures of demographic factors, depression symptom severity and comorbidity, stressful life events and service use in the YP (to allow a future economic evaluation) at baseline and follow-up. Questionnaires and interviews completed by the parent will provide information on baseline parental depression and the course of the parent’s depression throughout the study, plus parental anxiety and functional impairment.

### Data linkage

Consent will be sought from participants for anonymised data linkage to routine health and education records. This will allow longer-term follow-up in the future where treatment in health records would be the primary outcome.

### Process evaluation

A nested process evaluation (figure 2) will assess fidelity, adherence, feasibility and acceptability of the intervention and possible intervention mechanisms. It will also assess how key adaptations made to SWELL (ie, the addition of PTO) influence effectiveness and implementation (table 3). We aim to conduct focus groups with YP, parents and professionals involved in the youth-focused CBT intervention and PTO. We will additionally ask for feedback on trial processes which will include those allocated to both study arms (table 3). The sample size will be reviewed at each data collection phase to ensure data saturation has been reached and that no new themes emerge. We will complete a focus group/interviews with eight parents in PTO, the PTO professional team and the four CBT therapists. Participants will be purposively sampled to ensure maximum variation in terms of sex, age, ethnicity, educational level and outcome. Interviews and/or focus groups will be audio recorded, transcribed and de-identified.

**Table 3:** Nested process evaluation

	Skills for adolescent WELLbeing (SWELL) programme	Parent-treatment-optimisation (PTO)
Fidelity	Each therapist will audiorecord every group intervention session. For each intervention group, an experienced clinician (JA or DM) will rate one early group and one later group from the acute sessions (ie, 25%) using a fidelity checklist.	Qualitative interviews/focus groups will be conducted with professionals (psychiatrists, psychology assistants) involved in the PTO.
Adherence	The young people’s attendance and participation in group sessions will be recorded by therapists.	Parents’ attendance at the initial consultation and follow-ups will be recorded by a member of the treatment optimisation team.
Feasibility and acceptability	At 3-month follow-up, all young people in the intervention group and their parents will complete online questionnaires about their experience of receiving the intervention.Semistructured qualitative focus groups will be conducted with a subgroup of young people (n=16) and parents (n=8). Topic guides will ask about their experiences of receiving the intervention, with a focus on acceptability of online delivery and their views on potential implementation and scalability.There will also be qualitative interviews/focus groups with trial therapists (n=4) about their experiences of delivering the intervention and their perspectives on factors affecting future implementation.	At baseline, all parents who have received treatment optimisation will complete online questionnaires about their experience of receiving the intervention.Semistructured qualitative interviews/focus groups will be conducted with parents who received treatment optimisation (n=8) to explore the acceptability of this intervention.
Intervention mechanisms	The semistructured qualitative interviews/focus groups mentioned above will also help to examine underlying intervention mechanisms for the young person, including the CBT intervention for the young person and any perceived effect on family functioning or on the young person of PTO. We will seek the views of participating young people, parents and trial therapists and psychiatrists about what the active ingredients of the intervention might be. We will include prompts to assess active ingredients not captured in quantitative measures, including addressing stigma and non-specific mediators (eg, qualities of the group or the therapeutic alliance). We will also assess participant views of what helped and any potential barriers or implementation considerations.	
Experiences of and feedback on trial processes	Focus groups will be conducted with young people and parents about their experiences of trial processes. This will include the intervention groups described above and focus groups of young people (n=8) and parents (n=8) allocated to the control arm.	

In addition to exploring the SWELL intervention and PTO fidelity, adherence, feasibility and acceptability and intervention mechanisms, semistructured qualitative interviews/focus groups will be conducted with young people and parents randomised to the usual care arm regarding their experience of trial processes as part of the nested process evaluation.

### Data management

Data will be kept on a REDCap database held on a secure server at Cardiff University. Participant data will be encrypted, time-stamped and pseudoanonymised using an identification number. Only the researchers will have the information needed to translate the identification number to the participant identity.

### Analyses

The flow of participants through the trial will be presented as a Consolidated Standards of Reporting Trials diagram. Baseline demographic data will be presented for all consented and randomised participants, and patterns of attrition will be examined.

The primary analysis will be time-to-event analysis of the primary outcome (time to the first depressive episode collected at 9-month follow-up) and will use the complete case dataset. However, multiple imputation will be considered as a sensitivity analysis to examine possible dropout bias in the treatment effect estimate. Consistent with clinical practice, depression in the YP will be defined as YP depression reported by either parent/YP with a planned sensitivity check for YP report only. A Cox regression model will be used to determine the difference between trial arms, the HR and 95% CI. We will investigate whether a random effect to account for the group delivery clustering in the intervention arm needs to be included in the primary model. If the effect of the clustering is negligible, this will be excluded. Assumptions of the model (eg, constant hazard) will be checked to ensure model validity. Prespecified subgroups to be compared descriptively include those defined by baseline characteristics of the YP (eg, comorbidity) and parents (eg, depression status at randomisation). External treatment use will also be compared descriptively across study arms, and its effect will be evaluated by including this as a time-varying covariate in the Cox model.

Possible effect modification (eg, by PTO response, baseline demographic factors) will be investigated by including an arm*modifier interaction term. Mediation analyses will examine the effects of potential mediators (eg, improved problem-solving skills, improved parent-child relationship) on the primary outcome via inclusion of the mediator predictor in the primary model.

A sensitivity check will explore the effect of changes to inclusion criteria on the effectiveness of the intervention (online supplemental material 4). Only 20 participants were consented prior to the implementation of these changes, meaning their inclusion is unlikely to influence the main study findings in a substantial way.

Repeated measures analysis will be used for secondary outcomes collected at 3 months and 9 months. Linear mixed effects regression will be used to account for clustering by group delivery in the intervention arm (partial effect model) and will include baseline values as covariates where appropriate. Where the assumptions of the linear regression model are not met, transformation or categorisation will be considered. Logistic regression models will be used for binary outcomes. Main group effects and 95% CIs will be presented for all outcomes.

Qualitative data analysis will use a thematic analysis approach, a process of identifying, analysing, reporting and interpreting patterns or themes.^48^ Codes will be applied to broad themes and transcripts will be examined to identify key themes and subthemes. To ensure validity, a proportion of transcripts (approximately 20%) will be double-coded by a second qualitative researcher, and any discrepancies resolved by discussion. Thematic analysis will be supported by NVivo software.

### Trial management

A TMG and an independent TSC have been assembled to monitor progress and provide advice. The TSC has agreed to play the role of a data monitoring committee. The TMG is composed of the PI, co-investigators and the SWELL research team at the Wolfson Centre and the Centre for Trials Research. The TSC comprises external experts on trials involving YP’s mental health, an independent statistician, a young person and a parent with lived experience of depression.

### Monitoring adverse events and safety protocol

In the YP eligible for this study, there is an inherent risk of self-harm and suicide. We have therefore defined expected adverse events *a priori* as suicidal thoughts with a plan, self-harm or suicide attempts. These adverse events are not expected to be related to the intervention, but the additional contact with the study team for YP in the intervention arm may create greater opportunity for reporting adverse events. Adverse events reporting procedures will be followed by all researchers working on the trial. A standard study procedure for documenting and dealing appropriately with these events will be followed. This includes risk assessment, signposting to services and liaison with clinical services as needed (see online supplemental material 5 for procedure).

## Discussion

This is a large-scale RCT to examine the effectiveness of an evidence-based group CBT intervention, adapted for online delivery with an added parent treatment phase to prevent the onset of MDD in YP. Treatment optimisation will be offered for parents who have elevated depressive symptoms at study entry to improve the effectiveness of the intervention for the YP. Although an intergenerational intervention, it involves working separately with parents and YP to improve their mental health, an approach that is consistent with current UK systems for supporting mental health. An embedded process evaluation will investigate the impact of this key adaptation.

This study will also evaluate the mechanisms through which the intervention has its effect using hypothesis-driven quantitative methods as well as exploratory qualitative methods, providing important insights into how intergenerational interventions might work to prevent depression in YP.

The source intervention for YP has been shortened and will be delivered entirely online by therapists at a level equivalent to those working with low-intensity psychological interventions in the NHS, making it a much-needed,^12^ scalable prevention programme for depression, if found to be effective.

### Ethics and dissemination

Ethical approval was granted by Wales NHS Research Ethics Committee 5 (Wales.REC5@Wales.nhs.uk) before the trial commenced (IRAS 305331; REC 22/WA/0254). This manuscript is based on V.5.7 of the protocol dated 17 January 2025. Significant protocol modifications will be communicated by submitting an amendment for ethical review and updating trial registry details. We will share deidentified individual participant-level data following publication of the main trial results on Cardiff University’s research data repository. We will follow BMJ regulations on authorship. Trial results will be published in peer-reviewed journals.

### Trial status

Recruitment started on 21 August 2023, the first participant was consented on 19 September 2023 and 267 participants had been recruited by 02 June 2025. Recruitment is planned to be completed by August 2025 with analysis and dissemination between September 2025 and February 2027. Cardiff University is the study sponsor (ref: SPON1902-22) and the study contact for enquiries is SWELL@Cardiff.ac.uk.

## Supplementary material

10.1136/bmjopen-2025-100692 online supplemental file 1

10.1136/bmjopen-2025-100692 online supplemental file 2

10.1136/bmjopen-2025-100692 online supplemental file 3

10.1136/bmjopen-2025-100692 online supplemental file 4

10.1136/bmjopen-2025-100692 online supplemental file 5
