# Supplementary material for: Skills for adolescent WELLbeing (SWELL): protocol for a preventive effectiveness randomised controlled trial for young people at high-familial risk of depression with treatment optimisation for parents with depression at study entry comparing online group cognitive behavioural therapy (CBT) with treatment as usual
Source: BMJ Open. 2025 Jun 19;15(6):e100692. doi: 10.1136/bmjopen-2025-100692 (PMC12182113; doi:10.1136/bmjopen-2025-100692)
Supplement: online supplemental file 2 [file bmjopen-15-6-s002.docx]

## Supplemental Material 2

Participants will be randomised 1:1 using random permuted blocks stratified by site, with the unit of randomisation being the young person. The allocation schedule has been generated by the trial statistician by using ralloc (a STATA module to design randomised controlled trials) in STATA v17. The randomisation will be online to maintain allocation concealment from the trial recruiters and built into trial database. The randomisation system will be developed and tested by the Centre for Clinical Trials Research (Cardiff University) according to their standardised procedures.
