# Supplementary material for: Skills for adolescent WELLbeing (SWELL): protocol for a preventive effectiveness randomised controlled trial for young people at high-familial risk of depression with treatment optimisation for parents with depression at study entry comparing online group cognitive behavioural therapy (CBT) with treatment as usual
Source: BMJ Open. 2025 Jun 19;15(6):e100692. doi: 10.1136/bmjopen-2025-100692 (PMC12182113; doi:10.1136/bmjopen-2025-100692)
Supplement: online supplemental file 3 [file bmjopen-15-6-s003.docx]

Supplementary Material 3

Participant Consent Forms

| ID number (for researcher): | |
| --- | --- |
| **Consent form for parent/guardian participating in research** (Version 7.0,5^th^ March 2025) | |
| **Study title: A Randomised Controlled Trial of a group CBT intervention for young people with parental depression treatment optimisation: Skills for adolescent WELLbeing (SWELL)**  The researcher will read each of the consent form statements and will ask the participant for verbal consent after each statement. | |
| Check the box next to each statement to indicate that each statement has been  understood by each participant. | **Initials:** |
| 1. I have read and understood the information sheet for parent/guardian participating in research, dated 5^th^ March 2025 (version 5.9) for the above study. I have had the opportunity to ask questions and have had these answered satisfactorily. |  |
| 2. I understand that my participation is voluntary. I understand that I am free to withdraw at any time, without giving any reason, and without my health care or legal rights being affected. I understand that I can contact the researchers to let them know I would like to stop taking part. |  |
| 3. I agree to take part in this study by completing online questionnaires and interviews, to consider attending online consultations with the trial psychiatrist if I am identified as being currently depressed, and to consider attending an 8 week course of guided, online cognitive behavioural therapy (CBT) if the psychiatrist initiates this for me. |  |
| 4. I agree for online study assessments (including online interviews) to be audio recorded for research purposes only. |  |
| 5. I understand that all identifying information that I provide will be kept confidential and will be seen only by study researchers and regulators whose job it is to check the work of researchers. |  |
| 6. I agree for the data collected from me as part of this trial (with identifying information removed) to be used in the future by researchers in the UK and abroad. I understand that this research may involve use of the commercial sector. |  |
| 7. I agree that what I say during the project may be quoted word for word in reports, presentations and publications, but my personal information will not be revealed. |  |
| 8. I agree for my attendance at online consultations with the trial psychiatrist and my use of the online CBT course to be monitored for research purposes only. |  |
| 9. I agree to the way my data and information will be processed and stored, for up to 15 years in approved archiving facilities, following General Data Protection Regulation (GDPR). |  |
| 10. I understand that if I withdraw from the study, the data collected up to that point will be retained and used for the remainder of the study. |  |
| 11. I understand my GP will be informed that I am taking part in this study. Two other situations where the research team might contact my GP include (1) if they have a serious concern (e.g. if I describe thoughts of harming myself or someone else), (2) If I attend a consultation with the trial psychiatrist, the psychiatrist will write to my GP (and to me) updating the GP on their assessment and any suggested treatments. |  |
| **12. I agree to take part in the study** |  |
| **These are optional:** |  |
| 13. I agree to be contacted by the research team in the future about the possibility of taking part in another research project. |  |
| 14a. I agree to take part in a meeting to discuss my feedback on participating in the trial. |  |
| 14b. I understand that this meeting will be audio/video recorded for research purposes only, and the audio recordings may be transcribed by an external company. |  |
| 15. I agree that my study data can be securely linked to routinely collected datasets (such as those held in the Secure Anonymised Information Linkage (SAIL) databank) - for health research purposes only. I understand that all data will be anonymised and that I will not be identified. |  |
| 16. I would like to receive the general results of the study at the end of the project. |  |

Name of participant: Date: Signature:

Name of researcher taking consent: Date: Signature:

Lead researcher: Professor Frances Rice, Wolfson Centre for Young People’s Mental Health, Division of Psychological Medicine & Clinical Neuroscience, Cardiff University, Hadyn Ellis Building, Maindy Road, Cardiff CF24 4HQ. Email: [SWELL@cardiff.ac.uk](mailto:SWELL@cardiff.ac.uk)

| ID number (for researcher): | |
| --- | --- |
| **Consent form for parent/guardian of young person participating in research**  (Version 7.0 5^th^ March 2025) | |
| **Study title: A Randomised Controlled Trial of a group CBT intervention for young people with parental depression treatment optimisation: Skills for adolescent WELLbeing (SWELL)**  The researcher will read each of the consent form statements and will ask the participant for verbal consent after each statement. | |
| Check the box next to each statement to indicate that each statement has been  understood by each participant: | **Initials:** |
| 1. I have read and understood the information sheet for parent/guardian (of young person participating in research) dated 5^th^ March 2025 (version 5.9) for the above study. I have had the opportunity to ask questions and have had these answered satisfactorily. |  |
| 2. I understand that the participation of my child is voluntary. I understand that they are free to withdraw at any time, without giving any reason, and without their health care or legal rights being affected. I understand that they can contact the researchers to let them know they would like to stop taking part. |  |
| 3. I agree for my child to take part in this study by attending and participating in the online group cognitive behavioural therapy (CBT), completing any homework that is set for them, responding to texts that check in on their mood, and by completing online interviews and questionnaires. |  |
| 4. I understand that if my child is allocated to the online group CBT sessions, they will be audio-recorded for quality monitoring purposes. |  |
| 5. I agree for online study assessments (including online interviews) with my child to be audio recorded for research purposes only. |  |
| 6. I understand that all identifying information that my child provides will be kept confidential. I understand this will be seen only by study researchers and regulators whose job it is to check the work of researchers. |  |
| 7. I agree for the data collected from my child as part of this trial (with identifying information removed) to be used in the future by researchers in the UK and abroad. I understand that this research may involve use of the commercial sector. |  |
| 8. I agree that what my child says during the project may be quoted word for word in reports, presentations and publications, but their personal information will not be revealed. |  |
| 9. I agree for my child’s attendance and participation in the online group CBT and their completion of homework to be monitored for research purposes only. |  |
| 10. I agree to the way my child’s data and information will be processed and stored, for up to 15 years in approved archiving facilities, following relevant General Data Protection Regulation (GDPR). |  |
| 11. I understand that if my child withdraws from the study, the data collected up to that point will be retained and used for the remainder of the study. |  |
| 12. I understand my child’s GP will be informed that they are taking part in this study. The research team might also contact my child’s GP if they have a serious concern during this project (e.g. if my child expresses thoughts of harming themselves or someone else). |  |
| **13. I agree for my child to take part in the study** |  |
| **These are optional:** |  |
| 14. I agree for me and my child to be contacted by the research team in the future about the possibility of taking part in another research project. |  |
| 15a. I agree for my child to take part in a meeting to discuss their feedback on the study and/or the online group CBT. |  |
| 15b. I understand that this meeting with my child will be audio/video recorded for research purposes only, and the audio recordings may be transcribed by an external company. |  |
| 16. I agree that my child’s study data can be securely linked to routinely collected datasets (such as those held in the Secure Anonymised Information Linkage (SAIL) databank) - for health research purposes only. I understand that all data will be anonymised and that my child will not be identified. |  |
| 17. I would like my child to receive the general results of the study at the end of the project. |  |

Name of participant: Date: Signature:

Name of researcher taking consent: Date: Signature:

Lead researcher: Professor Frances Rice, Wolfson Centre for Young People’s Mental Health, Division of Psychological Medicine & Clinical Neuroscience, Cardiff University, Hadyn Ellis Building, Maindy Road, Cardiff CF24 4HQ. Email: [SWELL@cardiff.ac.uk](mailto:SWELL@cardiff.ac.uk)

|  | **Assent form for young person** (Version 7.0, 5^th^ March 2025) ID number (for researcher): | | |
| --- | --- | --- | --- |
|  | **Study title: A Randomised Controlled Trial of a group CBT intervention for young people with parental depression treatment optimisation: Skills for adolescent WELLbeing (SWELL)**  **The researcher will read each of the assent form statements and will ask the participant for verbal assent after each statement.** | | |
| Check the yes/no box next to each statement to indicate that each statement has been  understood by each participant | | Yes | No |
| 1. Have you read (or had read to you) the information sheet for young people? | |  |  |
| 2. Do you understand what this project is about? | |  |  |
| 3. Have you had the chance to ask questions? | |  |  |
| 4. Have your questions been answered in a way you understand? | |  |  |
| 5. Do you understand that it’s OK to stop taking part at any time? Do you understand that you can let the researchers know that you would like to stop taking part? | |  |  |
| 6. Do you understand that if you are allocated to the online group cognitive behavioural therapy (CBT) sessions, they will be audio-recorded? | |  |  |
| 7. Are you willing to attend and participate in the online group CBT, complete any homework that is set for you, respond to texts that check in on your mood, and to complete interviews and questionnaires? | |  |  |
| 8. Do you understand that study assessments (including online interviews) will be audio-recorded for research purposes only? | |  |  |
| 9. Do you understand that all identifying information you give will be kept private? Do you understand it will be seen only by study researchers and people whose job it is to check the work of researchers? | |  |  |
| 10. Do you understand that the data collected from you as part of this trial (with identifying information removed) may be used in the future by researchers in the UK and abroad, and that this research may involve use of the commercial sector? | |  |  |
| 11. Do you understand that what you say during the project may be used word for word in reports, presentations and publications, but your personal information will not be revealed? | |  |  |
| 12. Do you agree for your attendance and participation in the online group CBT and completion of homework to be monitored for research purposes only? | |  |  |
| 13. Do you agree that your details and information will be processed and stored, for up to 15 years in approved archiving facilities (e.g. secure computers), following General Data Protection Regulation (GDPR)? | |  |  |
| 14. Do you understand that if you stop taking part, the information you have already given will be saved and used for the rest of the study? | |  |  |
| 15. Do you understand that the study team will contact your family doctor (GP) to let them know you are taking part in the study? | |  |  |
| 16. Do you understand that the study team might also contact your family doctor (GP) if they have a serious concern during this project (e.g. if you expressed thoughts of harming yourself or someone else)? | |  |  |
| **17. Do you agree to take part in the study?** | |  |  |
| **These are optional:** | |  |  |
| 18. Do you agree to be contacted by the research team in the future about taking part in another research project? | |  |  |
| 19a. Do you agree to take part in a meeting about your feedback on the study and/or the online group? | |  |  |
| 19b. Do you understand that if you participate in the feedback meeting, it will be audio/video recorded for research purposes only, and that the audio recordings may be transcribed by an external company? | |  |  |
| 20. Do you agree that your data collected as part of this study can be linked to other datasets (sets of information) that are collected routinely about you, such as your health records? Do you understand that this will be for research purposes only, and it will not be possible to work out who took part in the study? | |  |  |
| 21. I would like to receive the general results of the study at the end of the project. | |  |  |

**PLEASE CONTINUE ONTO THE NEXT PAGE**

Participant name: Date: Signature:

Parent/guardian name: Date: Signature:

Researcher name: Date: Signature:

Lead researcher: Professor Frances Rice, Wolfson Centre for Young People’s Mental Health, Division of Psychological Medicine & Clinical Neuroscience, Cardiff University, Hadyn Ellis Building, Maindy Road, Cardiff CF24 4HQ. Email: [SWELL@cardiff.ac.uk](mailto:SWELL@cardiff.ac.uk)

ID number (for researcher):

|  | **Consent form for young person age 16-19** (Version 4.0, 5^th^ March 2025) | |
| --- | --- | --- |
|  | **Study title: A Randomised Controlled Trial of a group CBT intervention for young people with parental depression treatment optimisation: Skills for adolescent WELLbeing (SWELL)**  The researcher will read each of the consent form statements and will ask the participant for verbal consent after each statement. | |
| Check the box next to each statement to indicate that each statement has been  understood by each participant | | **Initials:** |
| 1. I have read and understood the information sheet for young people dated 5^th^ March 2025 (version 5.8). I have been able to ask questions, and they have been answered satisfactorily. | |  |
| 2. I understand that it is up to me whether or not to take part. I can stop taking part at any time, without giving any reason, and without my health care or legal rights being affected. I understand that I can contact the researchers to let them know I would like to stop taking part. | |  |
| 3. I understand that if I am allocated to the online group cognitive behavioural therapy (CBT) sessions, they will be audio-recorded. | |  |
| 4. I agree to take part in this study by attending and participating in the online group CBT, completing any tasks that are set for me, responding to texts that check in on my mood, and completing interviews and questionnaires. | |  |
| 5. I agree for study assessments (including online interviews) to be audio-recorded for research purposes only. | |  |
| 6. I understand that all identifying information that I provide will be kept private and will be seen only by study researchers and regulators whose job it is to check the work of researchers. | |  |
| 7. I agree for the data collected from me as part of this trial (with identifying information removed) to be used in the future by researchers in the UK and abroad. I understand that this research may involve use of the commercial sector. | |  |
| 8. I agree that what I say during the project may be quoted word for word in reports, presentations and publications, but my personal information will not be revealed. | |  |
| 9. I agree for my attendance and participation in the online group CBT and completion of any tasks to be monitored for research purposes only. | |  |
| 10. I agree that my details and information will be processed and stored, for up to 15 years in approved archiving facilities (e.g. secure computers), following General Data Protection Regulation (GDPR). | |  |
| 11. I understand that if I stop taking part, the information I have already given will be saved and used for the rest of the study. | |  |
| 12. I understand that the study team will contact my family doctor (GP) to let them know I am taking part in the study. | |  |
| 13. I understand that the study team might also contact my family doctor (GP) if they have a serious concern (e.g. if I expressed thoughts of harming myself or someone else). | |  |
| **14. I agree to take part in the study** | |  |
| **These are optional:** | |  |
| 15. I agree to be contacted by the research team in the future about taking part in another research project. | |  |
| 16a. I agree to take part in a meeting to discuss my feedback on the study and/or the online group CBT. | |  |
| 16b. I understand that if I participate in the feedback meeting, it will be audio/video recorded for research purposes only, and that the audio recordings may be transcribed by an external company. | |  |
| 17. I agree that my study data can be securely linked to routinely collected datasets (such as those held in the Secure Anonymised Information Linkage (SAIL) databank) - for health research purposes only. I understand that all data will be anonymised and that I will not be identified. | |  |
| 18. I would like to receive the general results of the study at the end of the project. | |  |

**PLEASE CONTINUE ONTO THE NEXT PAGE**

If you want to take part, please write your name and today’s date:

Name of participant: Date: Signature:

Name of researcher taking consent: Date: Signature:

Lead researcher: Professor Frances Rice, Wolfson Centre for Young People’s Mental Health, Division of Psychological Medicine & Clinical Neuroscience, Cardiff University, Hadyn Ellis Building, Maindy Road, Cardiff CF24 4HQ. Email: SWELL@cardiff.ac.uk
