# Supplementary material for: Skills for adolescent WELLbeing (SWELL): protocol for a preventive effectiveness randomised controlled trial for young people at high-familial risk of depression with treatment optimisation for parents with depression at study entry comparing online group cognitive behavioural therapy (CBT) with treatment as usual
Source: BMJ Open. 2025 Jun 19;15(6):e100692. doi: 10.1136/bmjopen-2025-100692 (PMC12182113; doi:10.1136/bmjopen-2025-100692)
Supplement: online supplemental file 4 [file bmjopen-15-6-s004.docx]

Supplementary Material 4

Overview of changes made to trial since study opened

A summary of the changes made since the trial opened involved broadening inclusion criteria, which would better reflect how the intervention would be delivered in practice. We provide full details of the changes made, their timing and the involvement of our independent Trial Steering Committee (TSC) in making these changes below.

Recruitment to the SWELL trial opened on 21st August 2023 and was initially slow. We therefore focused on discussing recruitment at the first TSC meeting after opening recruitment as recommended (Avery et al., 2017). This occurred on 16^th^ November 2023. At that meeting, we agreed to try several new recruitment avenues (e.g. schools, other existing cohorts) and closely monitor the effect this had on expressions of interest and recruitment. During this period recruitment continued to be slow but appeared to be improving due to inclusion of these new recruitment methods. We continued to closely monitor recruitment figures and noticed a low transition rate from screening to eligibility (25%). We therefore further sought to try and improve our transition rate from screening to eligibility as recommended (Wilson et al., 2018). We had primarily based our initial inclusion and exclusion criteria on those used in the study by Garber et al (2009). At the next TSC meeting on 21^st^ March 2024 therefore we discussed a number of fairly minor alterations to the inclusion and exclusion criteria that we felt were pragmatic and could be justified scientifically and/or based on common practice in UK clinical services that would make the study more inclusive. Some minor changes were also made to simplify the criteria allowing the research team to screen interested parents and young people as efficiently as possible. We made these changes with a view to improve the ratio of those eligible and ineligible at screening. The amendments made were as follows:

- - We removed the exclusion criteria of PTSD in the parent. The rationale for this was that it was unlikely to impact on the treatment options as part of the treatment optimization (Ramanuj et al., 2019; NICE, 2022) and we know that depression is frequently comorbid (Ramanuj et al., 2019).
  - We simplified the requirement that the parent was willing to engage in parent treatment optimization to “willing to consider” parent treatment optimization.
  - We removed the exclusion criteria that the young person had completed a full course of Cognitive Behavioural Therapy (CBT) in the past. The rationale for this was that the SWELL intervention is intended as both a first episode and a relapse prevention program. Moreover, independent members of the TSC highlighted difficulties in knowing what may have constituted a prior course of CBT and whether it was evidence-based.
  - We simplified the requirement for the parent’s prior depression to at least one episode of major depressive disorder (as opposed to requiring at least 2 episodes of prior depression). The rationale for this was that any episode of depression in parent increases risk of depression in young people (Uher et al., 2023).
- We reduced the clinical-cut point for current depressive symptoms in the young person using the CES-D from >=20 to >=16. The rationale for doing this was that evidence shows that scores of >=16 represent moderate depression (Thapar et al., 2022; Stockings et al., 2015) and that the study aims to prevent depressive disorder from developing.
- Following the advice of our TSC, we extended the age-range of young people to 13-19 years (as opposed to 13-17 years). The rationale for this was that this reflects the definition of adolescence (Sawyer et al., 2012), rates of depression increase in late adolescence (Solmi et al., 2022) and that prior other early-intervention studies using similar types of intervention have included young people up to the age of 19 years (Hetrick et al., 2016)

We intend to include participants consented prior to these changes in the main analysis. However, we will undertake a sensitivity analysis to explore any effect of changes to criteria on the effectiveness of the intervention. Please note that the number of participants consented prior to these changes being implemented is low. Only 20 participants were consented prior to these changes being implemented and submitted as a non-substantial amendment to the ethics committee (non-substantial amendment 9, 1^st^ May 2024). Therefore, this is unlikely to impact in a substantial way on the main study findings.

A final change included updates to assumptions of the power estimation. The sample size estimation did not change following recruitment and while we made the decisions to change the assumptions of the power estimate before recruitment began, we updated the protocol via a non-substantial amendment after recruitment opened. The following updates were required: we updated the estimation to reflect the fact that the primary outcome at 9 month-follow-up is measured retrospectively. We had originally performed calculations assuming a conservative attrition rate at 9-month follow-up (of 20%). We updated this assumption to 10% because this figure more accurately reflects the rate seen in samples of this kind (e.g. Garber et al., 2009; Mars et al., 2012). We also updated the test to a one-sided test based on a previous observation of effectiveness in the Garber et al (2009) study. This information is reflected in version 5.7 of the study protocol submitted to IRAS and in the statistical analysis plan.

References

Avery KN, Williamson PR, Gamble C, O'Connell Francischetto E, Metcalfe C, Davidson P, Williams H, Blazeby JM; members of the Internal Pilot Trials Workshop supported by the Hubs for Trials Methodology Research. Informing efficient randomised controlled trials: exploration of challenges in developing progression criteria for internal pilot studies. BMJ Open. 2017 Feb 17;7(2):e013537. doi: 10.1136/bmjopen-2016-013537. PMID: 28213598; PMCID: PMC5318608.

Depression in adults: treatment and management NICE guideline [Internet]. 2022. Available from: [www.nice.org.uk/guidance/ng222](http://www.nice.org.uk/guidance/ng222)

Hetrick SE, Cox GR, Witt KG, Bir JJ, Merry SN (2016). Cognitive behavioural therapy (CBT), third-wave CBT and interpersonal therapy (IPT) based interventions for preventing depression in children and adolescents. Cochrane Database of Systematic Reviews 2016, Issue 8. Art. No.: CD003380. DOI: 10.1002/14651858.CD003380.pub4.

Mars B, Collishaw S, Smith D, Thapar A, Potter R, Sellers R, Harold GT, Craddock N, Rice F, Thapar A. Offspring of parents with recurrent depression: which features of parent depression index risk for offspring psychopathology? J Affect Disord. 2012 Jan;136(1-2):44-53. doi: 10.1016/j.jad.2011.09.002. Epub 2011 Oct 1. PMID: 21962850.

Ramanuj P, Ferenchick EK, Pincus HA. Depression in primary care: part 2—management. BMJ (Online). 2019;365:l835–l835.

Sawyer SM, Afifi RA, Bearinger LH, Blakemore SJ, Dick B, Ezeh AC, Patton GC. Adolescence: a foundation for future health. *Lancet* 2012; 379: 1630–40.

Solmi M, Radua J, Olivola M, Croce E, Soardo L, Salazar de Pablo G, Il Shin J, Kirkbride JB, Jones P, Kim JH, Kim JY, Carvalho AF, Seeman MV, Correll CU, Fusar-Poli P. Age at onset of mental disorders worldwide: large-scale meta-analysis of 192 epidemiological studies. Mol Psychiatry. 2022 Jan;27(1):281-295. doi: 10.1038/s41380-021-01161-7. Epub 2021 Jun 2. PMID: 34079068; PMCID: PMC8960395.

Stockings E, Degenhardt L, Lee YY, Mihalopoulos C, Liu A, Hobbs M, et al. Symptom screening scales for detecting major depressive disorder in children and adolescents: A systematic review and meta-analysis of reliability, validity and diagnostic utility. J Affect Disord. 2015;174:447–63.

Thapar A, Eyre O, Patel V, Brent D. Depression in young people. The Lancet (British edition). 2022;400(10352):617–31.

Uher R, Pavlova B, Radua J, Provenzani U, Najafi S, Fortea L, Ortuño M, Nazarova A, Perroud N, Palaniyappan L, Domschke K, Cortese S, Arnold PD, Austin JC, Vanyukov MM, Weissman MM, Young AH, Hillegers MHJ, Danese A, Nordentoft M, Murray RM, Fusar-Poli P. Transdiagnostic risk of mental disorders in offspring of affected parents: a meta-analysis of family high-risk and registry studies. World Psychiatry. 2023 Oct;22(3):433-448. doi: 10.1002/wps.21147. PMID: 37713573; PMCID: PMC10503921.

Wilson C, Rooshenas L, Paramasivan S, Elliott D, Jepson M, Strong S, Birtle A, Beard DJ, Halliday A, Hamdy FC, Lewis R, Metcalfe C, Rogers CA, Stein RC, Blazeby JM, Donovan JL. Development of a framework to improve the process of recruitment to randomised controlled trials (RCTs): the SEAR (Screened, Eligible, Approached, Randomised) framework. Trials. 2018 Jan 19;19(1):50. doi: 10.1186/s13063-017-2413-6. PMID: 29351790; PMCID: PMC5775609.
