# Supplementary material for: Skills for adolescent WELLbeing (SWELL): protocol for a preventive effectiveness randomised controlled trial for young people at high-familial risk of depression with treatment optimisation for parents with depression at study entry comparing online group cognitive behavioural therapy (CBT) with treatment as usual
Source: BMJ Open. 2025 Jun 19;15(6):e100692. doi: 10.1136/bmjopen-2025-100692 (PMC12182113; doi:10.1136/bmjopen-2025-100692)
Supplement: online supplemental file 5 [file bmjopen-15-6-s005.docx]

Supplementary Material 5

Suicide risk monitoring procedure

*If a study participant expresses suicidal thoughts a brief risk assessment is completed by the researcher and discussed with a trial clinician.*

*The risk assessment is based on answers to the following questions (included in the SWELL study risk assessment form below):*

*1. What thoughts have you had about ending your life?*

*2. Have you made any plans to end your life? If yes, please give details.*

*3. Have you taken any action?*

*4. What factors are stopping you from ending your life?*

*5. What support do you have?*

*6. Have you told anyone else about these thoughts? Does anyone else know?*

*Where current risk is identified, the researcher carrying out the risk assessment will discuss this with a trial clinician (psychiatrist/clinical psychologist). They will agree an action plan and the researcher will communicate it back to the participant. Where necessary a letter will be sent to the participant’s GP to make them aware of any concerns raised and the action plan discussed.*

*If it is not possible for the researcher to get in touch with a clinician and risk is thought to be high then they will contact the participant’s GP, agree an action plan and communicate it back to the participant.*

*If there is imminent risk to the participant (e.g., if without immediate intervention the young person/adult will likely come to harm) emergency services will be contacted (e.g., 999).  Researchers will inform the chief investigator at the earliest opportunity.*

**SWELL individual risk assessment form: suicidal ideation and intent**

| **Participant ID:** | | **Participant DOB:** | | | **Initials:** |
| --- | --- | --- | --- | --- | --- |
| **Screening ID:** | | | **Date of the event:**   \|  \|  \| / \|  \|  \| / \|  \|  \|  \|  \| \| --- \| --- \| --- \| --- \| --- \| --- \| --- \| --- \| --- \| --- \| | | |
| 1. **Who has expressed thoughts that necessitate an assessment of risk of suicidal intent?** | | | Parent  Young person | | |
| 1. **When were these expressed?** | | | During therapy session  During an assessment  At another point during the study  Please specify: | | |
| 1. **Record answers to the brief risk assessment** 2. What thoughts have you had about ending your life? | | |  | | |
| 1. Have you made plans to end your life? | | | Yes No    If yes, please give details: | | |
| 1. Have you taken any action? | | | Yes No  If yes, please give details: | | |
| 1. What factors are stopping you from ending your life? | | |  | | |
| 1. What support do you have? | | |  | | |
| 1. Have you told anyone about these thoughts? Does anyone else know? | | |  | | |
| 1. **Having completed the risk assessment form, do you believe the person to be actively suicidal?**   **If possibly or yes, or if you have any concerns, you must discuss with a clinician.** | | | Yes Possibly No | | |
| **5a. What plan did you agree? (Include details of how you communicated this to the participant).** | | |  | | |
| **5b. Name of clinician who reviewed this form: (must be a clinician from clinical team contact list below).** | | |  | | |
| **5c. Date clinician reviewed form:** | | | \|  \|  \| / \|  \|  \| / \|  \|  \|  \|  \| \| --- \| --- \| --- \| --- \| --- \| --- \| --- \| --- \| --- \| --- \| | | |
| **Name of person completing this form:** | **Date of completion:**   \|  \|  \| / \|  \|  \| / \|  \|  \|  \|  \| \| --- \| --- \| --- \| --- \| --- \| --- \| --- \| --- \| --- \| --- \| | | | **Signature:** | |
