# Supplementary material for: Skills for adolescent WELLbeing (SWELL): protocol for a preventive effectiveness randomised controlled trial for young people at high-familial risk of depression with treatment optimisation for parents with depression at study entry comparing online group cognitive behavioural therapy (CBT) with treatment as usual
Source: BMJ Open. 2025 Jun 19;15(6):e100692. doi: 10.1136/bmjopen-2025-100692 (PMC12182113; doi:10.1136/bmjopen-2025-100692)
Supplement: Supplementary file 6 [file bmjopen-2025-100692.reviewer_comments.pdf]

PEER REVIEW HISTORY

BMJ Open publishes all reviews undertaken for accepted manuscripts. Reviewers are asked to complete a checklist review form and are provided with free text boxes to elaborate on their assessment. These free text comments are reproduced below.

ARTICLE DETAILS

Title (Provisional)

Skills for adolescent WELLbeing (SWELL): protocol for a preventive effectiveness randomised controlled trial for young people at high-familial risk of depression with treatment optimisation for parents with depression at study entry comparing online group Cognitive Behavioural Therapy (CBT) to treatment as usual

Authors

Rice, Frances; Powell, Victoria; Eyre, Olga; Bevan Jones, R; Michelson, Daniel; Airdrie, Jac; Collishaw, Stephan; Thapar, Anita; Grozeva, Detelina; Munnery, Kim; Randell, Elizabeth; Brookes-Howell, Lucy; Garber, Judy; Thapar, Ajay; Moore, Graham; Harrison, Neil A; Playle, Rebecca; Bisson, Jonathan; McNamara, Rachel

VERSION 1 - REVIEW

**Reviewer**	**1**
**Name**	**Pan, Mei-Rong**
**Affiliation**	**Institute of Mental Health**
**Date**	**30-Apr-2025**
**COI**	**None**

General Comments

This protocol presents a methodologically robust randomised controlled trial (RCT) examining the preventive efficacy of an adapted online CBT intervention (SWELL) combined with parental treatment optimisation (PTO) for youth at high familial risk of depression. The integration of intergenerational components and digital delivery holds significant potential for scalability. While the study aligns with SPIRIT guidelines and includes a commendable process evaluation, the following revisions are recommended to enhance methodological transparency and scientific rigour.

Specific Comments

Introduction

Paragraph 2

The statement "YP with depressed parents are therefore a recognised high-risk group that could benefit from preventive interventions" requires stronger causal justification. Although the author provides support for individual premises, the synthesis showing how environmentally mediated effects translate the prevalence of parental depression into an increased risk of major depressive disorder (MDD) in offspring remains inadequately developed

STUDY OBJECTIVES

Intervention Adaptations

The rationale for reducing continuation sessions (from 6 to 3) is pragmatic but lacks empirical support. Please cite evidence that shorter continuation phases retain efficacy, particularly in preventive contexts.

Beseides, I'd like the authors to clarify how fidelity to the original CwS program will be maintained despite adaptations (e.g., therapist training, manualization). The mention of therapist competency assessments using ACCS is commendable but needs specifics (e.g., frequency of fidelity checks, criteria for remediation).

METHODS AND ANALYSIS

Sample Size

While the sample size calculation (N=400) references prior studies (e.g., Garber et al., 2009), the rationale for assuming a hazard ratio (HR) of 0.60 (vs. 0.63 in Garber) requires further justification. Please clarify whether this assumption accounts for the added PTO component or relies on speculative improvements. Also, please provide the exact parameters used in the STATA ART analysis (e.g., expected event rates, attrition assumptions) to ensure reproducibility.

Blinding

While assessor blinding is noted, the risk of performance bias (due to unblinded

participants/therapists) is underaddressed. I;d like the authors to discuss strategies to minimize bias in self-reported outcomes (e.g., standardized prompts, triangulation with parent reports).

Interventions

The PTO protocol (Table 2) is appropriately aligned with NICE guidelines but lacks detail on how treatment components (e.g., guided CBT, medication adjustments) are tailored to individual needs. Specify decision-making protocols for psychiatrists (e.g., criteria for escalating treatment).

Adherence metrics for PTO (completion of baseline + 4 follow-ups) seem lenient. Consider adding thresholds for engagement (e.g., module completion rates for online CBT).

Where will the intervention be delivered? It would be better to clarify.

Outcome Measures

For the depression risk score (Stephens et al., 2022), since it was developed for a median of 2 - 3 years risk prediction, briefly validate its relevance to the trial's 9 - month time frame. This could involve demonstrating how the score has been adapted or why it is still appropriate for shorter - term risk assessment in this study. For example, if there are specific sub - scores or factors within the depression risk score that are more relevant to the 9 - month period, explain how they will be used.

ETHICS AND DISSEMINATION

While ethical approval is noted, clarify procedures for managing adverse events (e.g., suicidality) in both trial arms, including escalation pathways and liaison with local mental health services.

This protocol describes a timely and impactful trial with potential to advance preventive strategies for adolescent depression. With revisions to address the above points, it will be suitable for publication in BMJ Open.

Additional comments

These are additional comments on the protocol, which should be discussed in the Discussion section.

1. The protocol permits participants to initiate new treatments during the trial, which may confound outcomes. Expand the statistical plan to explicitly adjust for external treatment use (e.g., as a time-varying covariate in Cox models).

2. Recruitment via existing cohorts and primary care may limit diversity (e.g., underrepresentation of underserved populations). Describe outreach strategies (e.g., social media advertising) to ensure a sociodemographically representative sample.

3. Please standardize terminology by consistently using either 'adolescents' or 'young people' throughout the manuscript, with explicit definition of the chosen term (e.g., WHO age range criteria).

This trial addresses a critical gap in youth depression prevention. By addressing the above points, the authors can strengthen the protocol's validity and ensure findings are both scientifically robust and clinically actionable.

Reviewer

2025.04.30

**Reviewer**	**2**
**Name**	**Hao, Ming**
**Affiliation**	**Gannan Medical University**
**Date**	**03-May-2025**
**COI**	**None**

The content studied in this article is very interesting. As is well known, the family has a significant impact on an individual's psychological well-being, and it is meaningful to improve the mental health of parents or children from a family perspective.

However, this paper needs to address many issues that may cause confusion for readers before publication.

Whether the intervention methods proposed by the author have truly been implemented through practice.

If practice is carried out, it should provide information on the depression levels or incidence rates of parents or children at baseline, after intervention, and other time points. This is the best way for readers to determine whether the method is effective.

The author has only provided a detailed introduction to this method at present. Although this method may seem reasonable, untested methods are more like research plans than papers.

VERSION 1 - AUTHOR RESPONSE

Reviewer: 1
Dr. Mei-Rong Pan, Institute of Mental Health, National Clinical Research Center for Mental Disorders
Comments to the Author:
General Comments
This protocol presents a methodologically robust randomised controlled trial (RCT) examining the preventive efficacy of an adapted online CBT intervention (SWELL) combined with parental treatment optimisation (PTO) for youth at high familial risk of depression. The integration of intergenerational components and digital delivery holds significant potential for scalability. While the study aligns with SPIRIT guidelines and includes a commendable process evaluation, the following revisions are recommended to enhance methodological transparency and scientific rigour.

Specific Comments
Introduction
Paragraph 2
The statement "YP with depressed parents are therefore a recognised high-risk group that could benefit from preventive interventions" requires stronger causal justification. Although the author provides support for individual premises, the synthesis showing how environmentally mediated effects translate the prevalence of parental depression into an increased risk of major depressive disorder (MDD) in offspring remains inadequately developed

Response: We believe this statement may have been distracting for the reviewer because there is no need to explore the potential causal mechanisms to justify the statement that the children of parents with depression are a well-recognised high-risk group meriting consideration for preventive interventions – multiple policy reports including the World Health Organisation Action Plan highlight the children of parents with mental illness as meriting special consideration for early intervention and prevention. Instead, we included the statement about environmentally mediated inter-generational transmission because it implies that this may be amendable to change. Nevertheless, there are several studies that point at potential explanatory processes in inter-generational transmission of depression, namely parent-child relationship quality and

shared stressors, and we consider these in our logic model. We have therefore responded to this reviewer comment in the manuscript by moving the original statement and adding information on the evidence of potential causal inter-generational environmental processes to page 12 where we describe our logic model and approach to mediators.

STUDY OBJECTIVES

Intervention Adaptations

The rationale for reducing continuation sessions (from 6 to 3) is pragmatic but lacks empirical support. Please cite evidence that shorter continuation phases retain efficacy, particularly in preventive contexts.

Response: In making this decision, we consulted with the original intervention developers and examined the data on dosage effects of therapeutic and preventive interventions (as currently outlined on Page 6). In brief, there is consistent evidence including from the original Coping with Stress program that there is variability in the number of sessions that participants attend and that efficacy of programs is retained provided participants at least partially complete the program (Dickerson et al., 2024; PMID: 38904429*). Thus, there is evidence for a negatively accelerating growth curve in youth treatment and prevention outcomes over time such that there are greater beneficial effects of earlier sessions (also see Stice et al., 2009; PMID: 19485590). We now cite this evidence in the manuscript on Page 6 which when combined with pragmatic decisions about implementation provides further justification of our decision to reduce the number of continuation sessions. Following consultation with the original intervention developers, our team made the decision that given the key purpose of the continuation sessions (to practice skills, trouble shoot difficulties and revise core skills) there was benefit from a pedagogical perspective to ensuring that the continuation sessions covered and built on core content delivered in the acute sessions. On page 10, we now additionally clarify that the original CwS developers were consulted during the process of adapting the intervention.*

Beseides, I'd like the authors to clarify how fidelity to the original CwS program will be maintained despite adaptations (e.g., therapist training, manualization). The mention of therapist competency assessments using ACCS is commendable but needs specifics (e.g., frequency of fidelity checks, criteria for remediation).

Response: As requested, further details have been included on page 10 and in Table 3 outlining the therapist training requirements and providing further detail on quality assurance requirements and supervision.

METHODS AND ANALYSIS

Sample Size

While the sample size calculation (N=400) references prior studies (e.g., Garber et al., 2009), the rationale for assuming a hazard ratio (HR) of 0.60 (vs. 0.63 in Garber) requires further justification. Please clarify whether this assumption accounts for the added PTO component or relies on speculative improvements. Also, please provide the exact parameters used in the STATA ART analysis (e.g., expected event rates, attrition assumptions) to ensure reproducibility.

Response: The effect size observed in the study by Garber et al (2009) was a hazard ratio (HR) of 0.63. We expect the effect of the intervention in this RCT to be slightly greater than 0.63 due to the addition of parent depression treatment before randomisation. We therefore define the minimum clinically important difference (MCID) for this study as an HR of at least 0.60 (equivalent to an 11% difference in MDD between study arms) and this HR is used in the sample size calculations. The exact parameters used in the STATA ART are the following: equal group sizes (2), follow-up period – 9 months, hazard ratio 0.60, expected event probabilities: 0.327 (control arm, based on Garber et al.) and 0.211 (in the intervention arm), one sided alpha (0.05), 10% loss of follow up. The additional information of expected event probabilities is now included in the sample size section on Page 8-9; (all other details on the parameters were included in the previously submitted version).

Blinding

While assessor blinding is noted, the risk of performance bias (due to unblinded participants/therapists) is underaddressed. I;d like the authors to discuss strategies to minimize bias in self-reported outcomes (e.g., standardized prompts, triangulation with parent reports).

Response: The primary outcome is assessed by blind-assessors using a criterion-based semi-structured clinical interview that assesses major depressive disorder. Using a measure where criteria are pre-specified and implemented by a trained assessor guards to some degree against the possibility of performance bias i.e. where there are systematic differences in the way adolescents report on outcomes because they are aware of their study arm allocation. As highlighted in the manuscript the following procedures are used to minimize bias in self-reports: assessors follow guidance during follow-up assessments to guard against participants disclosing study arm allocation. All instances of unintentional unblinding of assessors are logged and will be examined by the statistical team at the end of the study. All questionnaire assessments in the study include standardized prompts. We have reports from young people and from parents about young person outcomes which allows consistency of effects across raters to be examined. Therapists who are, of course, not blinded do not assess study outcomes. We now include a discussion of the possibility of performance bias and the attempts we make to address this issue in the manuscript on Page 9 where we say:

"We will mitigate this by utilising: an interviewer-led criterion-based clinical interview to define the primary outcome and standardized prompts for questionnaires. We will also evaluate consistency of effects for outcomes reported by different raters (YP and parents/carers)."

Interventions

The PTO protocol (Table 2) is appropriately aligned with NICE guidelines but lacks detail on how treatment components (e.g., guided CBT, medication adjustments) are tailored to individual needs. Specify decision-making protocols for psychiatrists (e.g., criteria for escalating treatment).

Adherence metrics for PTO (completion of baseline + 4 follow-ups) seem lenient. Consider adding thresholds for engagement (e.g., module completion rates for online CBT).

Where will the intervention be delivered? It would be better to clarify.

Response: The parent treatment optimisation (PTO) is not randomised and does not form part of the intervention for the young person. The rationale of the PTO is to provide good quality healthcare consistent with NICE guidelines in order to give the intervention for the young person the best chance of working (because current parental depression undermines the effectiveness of CBT preventive and therapeutic interventions for youth depression e.g. Garber et al 2009). We have added a statement for the rationale of PTO to clarify that it does not form part of the randomised intervention. As requested, we have re-written the description of PTO in the manuscript for clarification Page 9 and added more detail to Table 2.

In brief, all patients have a psychiatric assessment and are offered psychoeducation about depression, self-management options and a medication review. All patients are referred to guided CBT and are usually recommended to start one of the following programmes: 'Space from Depression' or 'Space from Depression and Anxiety'. Overall, this approach is consistent with NICE guidelines for depression in adults and NHS practice in the UK. After this initial assessment, recommendations are made to their GP (e.g. regarding medication changes).

All psychiatrists are highly trained (consultant or higher trainee level) and will use their clinical judgement regarding the treatment options (consistent with NICE guidelines). Therefore, there are no pre-specified escalation protocols, but typically the treatment would be escalated if there is no improvement in their symptoms (e.g. as shown in the PHQ scores) or if there is a deterioration or clinical concern (e.g. risk of self-harm or suicide). This is now included as a footnote to Table 2.

The purpose of the check-ins is to: check on progress in initiating suggested psychiatrist recommendations, to encourage patients to initiate and engage with the guided CBT and to check for adverse events (i.e. medication side effects or clinical risk namely deteriorating mental state and/or suicide risk). We plan to describe adherence to PTO descriptively in the study results. Given the rationale of PTO and the non-randomised nature of PTO, we do not consider it necessary to add extra thresholds for engagement. We include detail on where PTO is delivered in the manuscript on Page 9 (either at the CUPS clinic at Cardiff University or via video/phone call – most often via videocall).

Outcome Measures
For the depression risk score (Stephens et al., 2022), since it was developed for a median of 2 - 3 years risk prediction, briefly validate its relevance to the trial's 9 - month time frame. This could involve demonstrating how the score has been adapted or why it is still appropriate for shorter - term risk assessment in this study. For example, if there are specific sub - scores or factors within the depression risk score that are more relevant to the 9 - month period, explain how they will be used.

Response: We intend to use the risk score as originally developed. However, minor adaptations to the reporting period for the information collected to be included in the risk score will be made. The score will utilise information collected at 9-months and the reporting time-frame for the included measures allows this. The variables included in the risk score are: age, biological sex, emotional problems as assessed by the Strengths and Difficulties questionnaire (reporting time frame is past 6 months), low family income, stressful life events in the past 9 months, and parent PHQ-9 score >=10 (reporting time frame is past 2 weeks). These data will be assessed at the 9-month follow-up. Information on the reporting periods has been updated in Table 5.

ETHICS AND DISSEMINATION
While ethical approval is noted, clarify procedures for managing adverse events (e.g., suicidality) in both trial arms, including escalation pathways and liaison with local mental health services.

Response: The study has a standard procedure for dealing with identified clinical risk, including suicidal ideation. This now is mentioned in more detail on Page 15 of the manuscript and a summary of this process including the risk assessment is now included in Supplementary Material 5. In brief, if a study participant expresses suicidal thoughts, a brief risk assessment is completed by the researcher and discussed with a trial clinician. The risk assessment is based on answers to the following questions:

1. What thoughts have you had about ending your life?

2. Have you made any plans to end your life? If yes, please give details.

3. Have you taken any action?

4. What factors are stopping you from ending your life?

5. What support do you have?

6. Have you told anyone else about these thoughts? Does anyone else know?

Where current risk is identified, the researcher carrying out the risk assessment will discuss this with a trial clinician (psychiatrist/clinical psychologist). They will agree an action plan and the researcher will communicate it back to the participant. Where necessary a letter will also be sent to the participant's GP to make them aware of any concerns raised and the action plan discussed.

If it is not possible for the researcher to get in touch with a trial clinician and risk is thought to be high then the researcher will contact the participant's GP, agree an action plan and communicate it back to the participant.

If there is imminent risk to the participant (e.g., without immediate intervention the young person/adult will likely come to harm) emergency services will be contacted (e.g., 999). Researchers will inform the chief investigator at the earliest opportunity.

This protocol describes a timely and impactful trial with potential to advance preventive strategies for adolescent depression. With revisions to address the above points, it will be suitable for publication in BMJ Open.

Response: We thank the reviewer for this comment and hope the revisions made satisfactorily address the points made.

Additional comments
These are additional comments on the protocol, which should be discussed in the Discussion

section.

1. The protocol permits participants to initiate new treatments during the trial, which may confound outcomes. Expand the statistical plan to explicitly adjust for external treatment use (e.g., as a time-varying covariate in Cox models).

Response: This is currently mentioned in the strengths and limitations summary of the trial where we state: "For ethical and pragmatic reasons, participants are free to start any new treatment during the study period meaning that external treatment use must be evaluated as an explanation of any differences between trial arms." As requested, we have now included more detail in the statistical analysis plan on Page 14. We say: "External treatment use will also be compared descriptively across study arms and its effect will be evaluated by including this as a time-varying covariate in the survival model." Detailed information about this will be provided in the statistical analysis plan.

2. Recruitment via existing cohorts and primary care may limit diversity (e.g., underrepresentation of underserved populations). Describe outreach strategies (e.g., social media advertising) to ensure a sociodemographically representative sample.

Response: In England and Wales, UK, it is estimated that between 90% and 100% of the population is registered with a general practitioner, which is a primary method for recruitment for the SWELL study. We have sampled areas of England and Wales that include deprived populations and have included GP surgeries in these areas. Indeed, Wales includes some of the most deprived areas in the UK and we have worked to try and include ways of offering people living in these deprived areas the opportunity to take part (i.e. by sampling GP practices in these areas and putting up posters in locations in these areas). We have used social media outreach strategies as suggested but in practice, like other studies, have found that this increases misrepresentation by potential participants (French et al., 2025; https://doi.org/10.1016/S2215-0366(24)00359-6) and at least anecdotally does not appear to improve diversity. We have put up study posters in a range of community settings (e.g. leisure centres, health centres, libraries) to increase awareness of the study in a wide range of populations.

We include a statement on this in the recruitment section where we say: "To help ensure a diverse sample we will minimise inclusion criteria and recruit in socioeconomically diverse areas".

3. Please standardize terminology by consistently using either 'adolescents' or 'young people' throughout the manuscript, with explicit definition of the chosen term (e.g., WHO age range criteria).

Response: We now consistently use the term young people throughout. The age range of participants included in the trial (13-19 years) is consistent with WHO, UN and scientific definitions of adolescence. https://www.unicef.org/health/adolescent-health-and-well-being However, we retain the term young people and use this throughout because this term is considered more inclusive in the UK, is the preferred terminology by teenagers themselves, and allows us to concisely refer to the young person lived experience input into the design of the trial

which included young people aged 14-24 years. We include a definition and reference to support this on Page 5.

This trial addresses a critical gap in youth depression prevention. By addressing the above points, the authors can strengthen the protocol's validity and ensure findings are both scientifically robust and clinically actionable.

Response: We appreciate the reviewer comment that this trial addresses a critical gap in youth depression prevention. We have addressed the above points and the editor suggestions and believe these changes have improved the protocol.

Reviewer
2025.04.30

Reviewer: 2
Dr. Ming Hao, Gannan Medical University
Comments to the Author:
The content studied in this article is very interesting. As is well known, the family has a significant impact on an individual's psychological well-being, and it is meaningful to improve the mental health of parents or children from a family perspective.
However, this paper needs to address many issues that may cause confusion for readers before publication.
Whether the intervention methods proposed by the author have truly been implemented through practice.
If practice is carried out, it should provide information on the depression levels or incidence rates of parents or children at baseline, after intervention, and other time points. This is the best way for readers to determine whether the method is effective.
The author has only provided a detailed introduction to this method at present. Although this method may seem reasonable, untested methods are more like research plans than papers.

Response: We are gratified that the reviewer finds the content in the article interesting and meaningful. The intervention methods described in this protocol paper outline the processes we intend to implement to deliver and analyse the results from the planned randomised controlled trial. Publishing the protocol ahead of completing the trial is a standard part of conducting a clinical trial and is important for a range of reasons: a) the protocol outlines a priori the decision making and plans for analysis allowing the scientific community to evaluate whether the eventual analysis and results are consistent with what was originally intended and b) it adds accountability for authors to report results of the trial in a timely manner. Publication of protocols is also a key aspect of open science which is important for ensuring reporting transparency, research integrity and reproducibility. We plan to publish the results of the trial in a peer-reviewed journal once the trial and analysis is completed. For this reason, the results of the trial are not reported here but they will be reported in a separate publication once the trial is

completed and the data have been analysed.
